# Extent, transparency and impact of industry funding for pelvic mesh research: a review of the literature

**DOI:** 10.1186/s41073-024-00145-9

**Published:** 2024-04-30

**Authors:** Angela Coderre-Ball, Susan P. Phillips

**Affiliations:** 1 Centre for Studies in Primary Care, Queen’s University, Kingston, Canada; 2 Family Medicine and Public Health Sciences, Queen’s University, Kingston, Canada

**Keywords:** Pelvic mesh, Conflicts of interest, Industry funding, Research methods, Uterine prolapse, stress urinary incontinence, women's health

## Abstract

**Background:**

Conflicts of interest inherent in industry funding can bias medical research methods, outcomes, reporting and clinical applications. This study explored the extent of funding provided to American physician researchers studying surgical mesh used to treat uterine prolapse or stress urinary incontinence, and whether that funding was declared by researchers or influenced the ethical integrity of resulting publications in peer reviewed journals.

**Methods:**

Publications identified via a Pubmed search (2014–2021) of the terms mesh and pelvic organ prolapse or stress urinary incontinence and with at least one US physician author were reviewed. Using the CMS Open Payments database industry funding received by those MDs in the year before, of and after publication was recorded, as were each study’s declarations of funding and 14 quality measures.

**Results:**

Fifty-three of the 56 studies reviewed had at least one American MD author who received industry funding in the year of, or one year before or after publication. For 47 articles this funding was not declared. Of 247 physician authors, 60% received > $100 while 13% received $100,000-$1,000,000 of which approximately 60% was undeclared. While 57% of the studies reviewed explicitly concluded that mesh was safe, only 39% of outcomes supported this. Neither the quality indicator of follow-up duration nor overall statements as to mesh safety varied with declaration status.

**Conclusions:**

Journal editors’ guidelines re declaring conflicts of interest are not being followed. Financial involvement of industry in mesh research is extensive, often undeclared, and may shape the quality of, and conclusions drawn, resulting in overstated benefit and overuse of pelvic mesh in clinical practice.

## Introduction

When medical research and vested interest collide, objectivity, research integrity, and best clinical practices are sometimes the victims. Compromise to objectivity can arise via ghost management of research [1], that is by direct involvement of industry personnel, or indirectly through industry transfers of honoraria, gratuities, or speaker payments made to independent researchers [2]. Circumstances such as these, that “create a risk that judgments or actions regarding a primary interest will be unduly influenced by a secondary interest are defined as conflicts of interest (COI)” [3]. COI stemming from industry funding can, although do not always [4], bias design, recruitment, conduct, choice of outcome measures, or reporting, all of which have the potential to distort study findings and undermine medical practice [5–7]. The United States Centers for Medicare & Medicaid Services Open Payments [8] database documents any industry payment of at least $10 and annual payments of $100 or more made to American physician researchers since 2013. Its creation has facilitated identifying a portion of corporate support for medical research.

We wished to examine the extent, accuracy and implications of COI reporting among authors studying the effectiveness and safety of one particular medical device, pelvic mesh. The CMS Open Payments database described above enables this examination although only for authors who were or are US physicians. Surgical mesh was first used in hernia surgery in the 1950s [9] and has become the standard of care for hernia repairs, although controversy remains [10]. By the late 1990s, surgical mesh was routinely being inserted trans-vaginally to treat pelvic organ prolapse (POP) and stress urinary incontinence (SUI). This repurposing required no approval in the US because the Food and Drug Administration’s (FDA) 510k route grants automatic authorization for products deemed to be equivalent to predicate devices already in use [11, 12]. Prior to 1976 the FDA did not require testing of any biomedical devices, meaning surgical mesh had never undergone pre-market testing [13]. Studies of success, failure and safety of both hernia and pelvic mesh are, therefore, generally retrospective reviews tracking outcomes of use in patients.

The FDA estimates that one in eight women (in the US) undergo surgery to repair uterine prolapse [14]. Post-market evidence from peer-reviewed journals has generally endorsed pelvic mesh to be a successful treatment for POP and SUI [15]. At the same time there are reports from an unknown proportion of female mesh recipients questioning that success [16, 17]. Commentaries have noted the close links among industry, researchers, surgeons and professional organizations that examine or voice support for pelvic mesh use [18]. Two studies of mesh used for hernia repairs raise questions about the evidence supporting its success and safety in that setting. First, despite many accounts of the value of mesh for hernia repair, none has reported on women, specifically, or considered that women’s greater immune response to foreign materials might predispose to disproportionate harm from insertion of the product [12, 19]. Second, Sekigami and colleagues [20] determined that the majority of studies of mesh used for hernia repairs did not accurately report COI.

Whether and how industry funding is entwined with published research on pelvic mesh is unknown. As noted above, what is known is that such funding compromises medical research in general [21]. Our objectives were, therefore, to: (1) examine the scope of industry funding provided to US physician-authors of pelvic mesh research; (2) determine the proportions of that funding that were declared or undeclared and; (3) explore whether the methodologic strength and conclusions of industry funded studies differed from those without industry support.

## Methods

### Study selection/data extraction

We undertook a cross-sectional review of publications identified in a PubMed search in October 2021. All studies related to surgical mesh used in POP and SUI repairs were initially identified. Included were clinical trials and observational studies with at least one American physician author, and that examined post-surgical outcomes for polypropylene mesh inserted for the treatment of POP or SUI. We excluded studies with no original data, no US physician authors, those whose main purpose was to compare surgical techniques (e.g., single incision mesh vs. sacrospinous ligament fixation), studies using only autologous material or non-polypropylene mesh, and studies that only examined peri-operative outcomes.

Search terms included (POP[title/abstract] OR SUI[title/abstract]) AND mesh[title/abstract]. Studies published between January 1, 2014, and September 30, 2021 were included. This time frame matched available entries in the CMS Open Payments database (see below). We chose the year of publication rather than year of acceptance as not all studies documented their acceptance date. Included were studies from any peer-reviewed journal. One author (ACB) screened studies for inclusion/exclusion criteria, and, if questions arose, discussion occurred between the two authors.

For each study, we extracted the authors’ and journal’s names, the date of acceptance where available and of publication, conflict-of-interest statements, funding declarations, the study’s inclusion and exclusion criteria, the outcome scales or measures, outcomes, and follow-up duration. We also determined the journal’s impact factor (April 2022). This information for 10 randomly selected studies was independently abstracted by both authors who then discussed and compared results to ensure accuracy and consistency. One author then extracted data for the remaining 48 studies. These data were then reviewed by both authors, together (see Outcomes, below).

### Open payments

For each physician author in each study, we searched the CMS Open Payments database to collect information on the types of payments (general, research, associated funding, and ownership and investment) made from all drug and device companies, the US dollar amount of each payment, and the companies making the payments. We included all payments authors received during the year before, the year of, and the year following publication to best ensure that all author payments that could be related to a study were captured. Payments totaling less than $100 over the three years, were entered as ‘no payment’. Small payments can influence physicians’ research and clinical behavior, however such amounts were not included to avoid modest sums or gratuities received that were likely unrelated to research.

### Findings assessed

The key findings examined were the extent of industry funding of research and the dollar difference between declared and actual industry payment received. First we tallied the number of authors and papers with COI, whether declared or not. We then examined the declaration status of each author with a COI. This was recorded as no discrepancy if that COI was declared. We then counted how many authors made no declaration or declared that they did not have a COI and recorded each author’s total payment from all categories over the three years. We did not examine each journal’s declaration of COI requirements and authors’ compliance with these, nor could we determine whether aspects of authors’ declarations were redacted by specific journals.

To assess the strength of each study we examined the following. We determined the duration of patient follow-up post-surgery. This measure was chosen because complications from pelvic mesh continue to arise years after insertion. If studies did not explicitly state a mean or median follow-up in their results we accepted the follow-up duration as the timeframe indicated in the methods/design. If no measure or statement was present, this was left blank. The use of objective (e.g., POP-Q) and/or subjective (e.g., UDI-6, pain) scales and/or outcomes was tracked for each study. Critical appraisal of each included study was assessed using a purpose-built data extraction and appraisal tool (see Table 1) based on the Joanna Briggs Institute Checklist for Cohort Studies [22]. Fourteen questions appraised methodology including, for example, “*Are the authors conclusions supported by the findings*?” and “*Did the authors make a statement that mesh was safe to use*?” To ensure reliability both authors critically appraised each study independently and then reviewed and discussed all appraisals together to resolve differences and reach consensus. Evaluation of whether authors’ conclusions were supported by the findings (Table 1, question ‘n’) was decided based on review of all the quality dimensions and discussion between both authors. For example, if a study made a positive conclusion about the effectiveness of mesh, but only followed patients for a short time (e.g., less than 12 months) and without a comparison group, it would be given a score of “no” or “unclear” for question ‘n’. Authors were blinded to information about funding when these quality indicators were recorded. Only after appraising and recording the strength of each study was this information merged with funding data.

### Statistical analysis

Univariate analyses were used to determine the presence of study characteristics that aligned with discrepancy between declared and undeclared COI. Guided by previous research on COI of authors studying hernia mesh [20] we included impact factor (continuous), follow-up time (continuous), author’s role (e.g. first author, contributing author, senior author – categorical), and recommendations of mesh safety and effectiveness (categorical – yes/no). We report the difference in payments received between those that declared and did not declare COI. The relationship between categorical variables (e.g., author role) and the presence of undeclared COI was determined using Chi-Square testing. Logistic regression was used to determine the association of continuous variables (e.g., impact factor, follow-up time) with whether or not there was a discrepancy between reported and discovered COI (from CMS Open Payments).

## Results

**Fig. 1:**
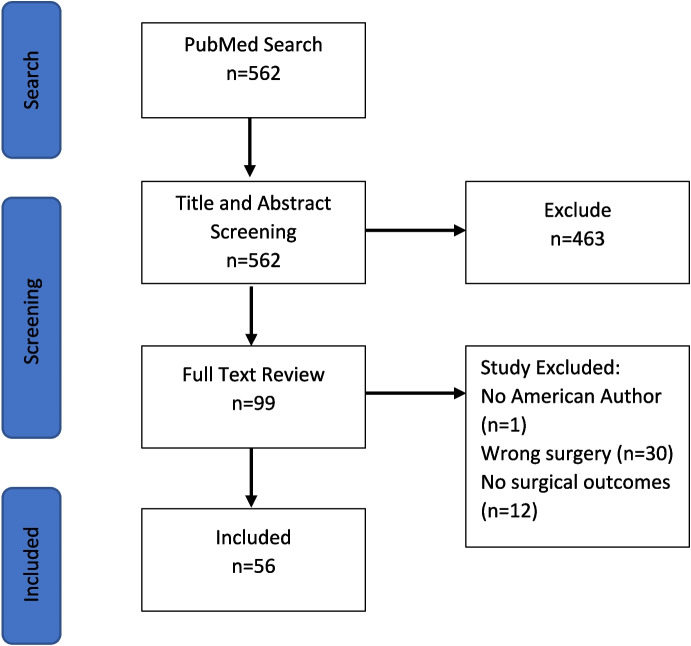
Overview of retrieved articles, screening and final included studies

Five hundred and sixty-two studies were retrieved from the PubMed search. After an initial review 56 of these were found to meet inclusion criteria (see Fig. 1: Overview of retrieved articles, screening process, and final included studies). The majority of the excluded studies had no author whose data would appear in Open Payments (i.e. no American physician author).

### Scope and declaration of industry funding: authors

There were a total of 299 authors of the 56 studies included in the full review. After excluding non-physicians and non-American physician authors as they would not be listed in the Open Payments database, 247 American MD authors remained and were included. For the remainder of the report, we only include these American MD authors in analyses.

Of the 247 authors and across all 56 included studies one hundred forty-nine authors (60%) received payments totaling more than $100. Eighty-one authors’ (33%) explicit declarations that they did not have COI aligned with Open Payments documentation of payments of less than $100 over the relevant three-year timeframe examined. An additional 12 authors (5%) made no declaration and did not receive payments totaling more than $100. Twenty-eight authors (11%) explicitly declared COI and did receive more than $100 in payments. One hundred and one authors (40%) explicitly declared that they had no COI but received payments, 20 (8%) did not make any declaration and received payments, and five authors (2%) declared a conflict although no payments were recorded in Open Payments.

Examining the dollar value of payments received, we found that the largest group receiving payments (36%, *n* = 54) was for amounts of between $100 and $1000 and was made to authors who did not declare any COI. The remaining undeclared payments were between $1,000-$10,000 (24%, *n* = 36), between $10,000-$100,000 (13%, *n* = 20) and >$100,000 (7%, *n* = 11).

**Fig. 2:**
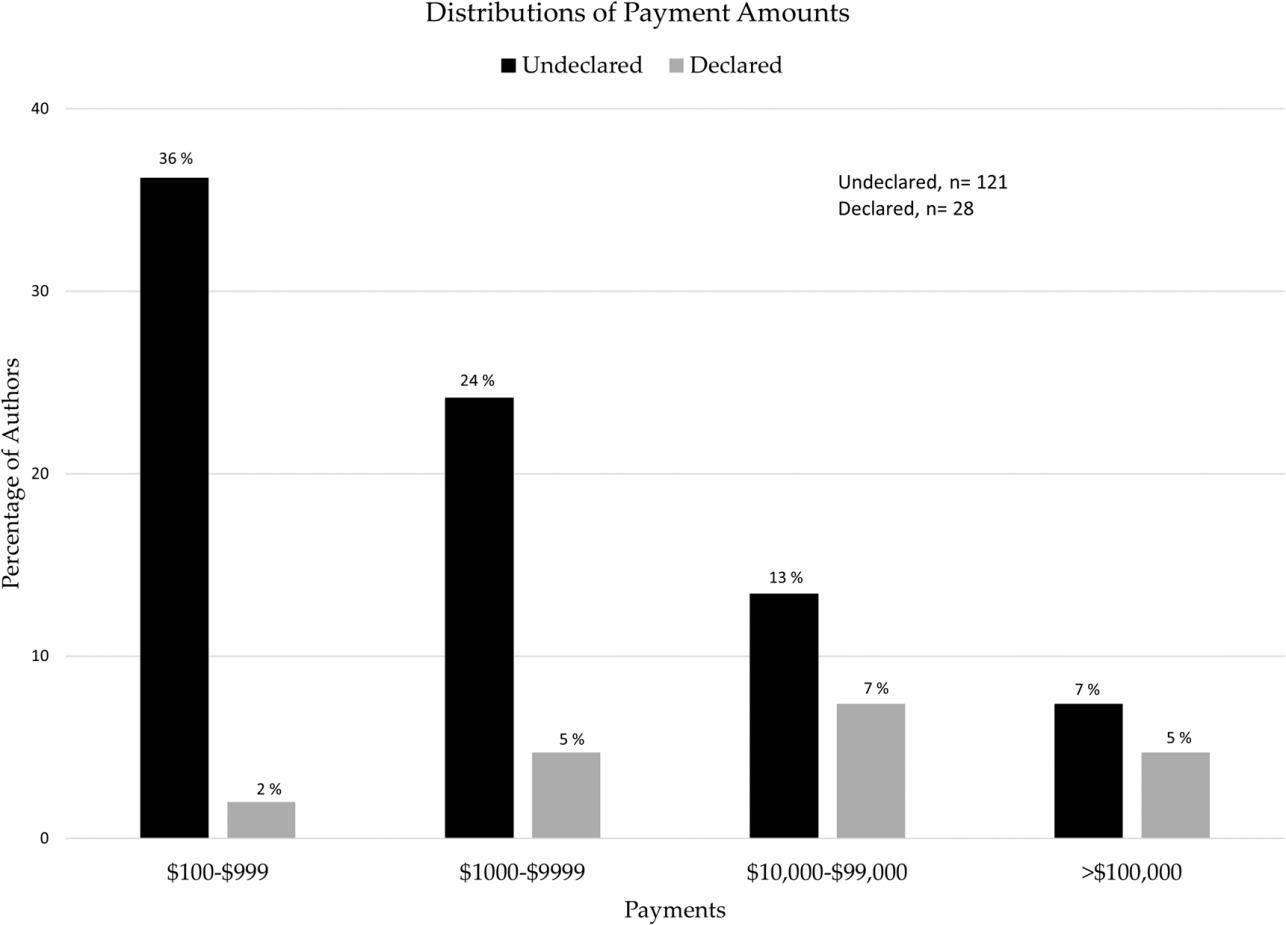
Proportions and amounts of declared and undeclared payments received by authors

The majority of payments for each of the four dollar amounts were undeclared (see Fig. 2: Proportions and amounts of declared and undeclared payments received by authors).

### Scope and declaration of industry funding: studies

Of the 56 studies reviewed, 53 (95%) had at least one American physician author with COI (declared or not). Thirty-nine (70%) included at least two American MD authors with COI, and 28 (54% of the 52 studies with 3 or more authors) had three or more American MD authors with COI.

Considering only non-declared COI, we found that 47 (84%) of studies included at least one American MD author with an undeclared COI, while 34 (61%) had at least two such authors, and 20 studies (38% of articles with more than 2 authors) had three or more authors with COI. Only three (5%) studies had no physician authors with any conflicts of interest (declared or not).

### Study characteristics aligned with undeclared COI

We next examined alignment of the dollar amount of industry funding received and any of the following: declaring a COI; the duration of follow-up in a study; or the journal’s impact factor.

The median payment for US authors was $18,678 (IQR ~ $5000-$99,000) for those with declared COI and $158 (IQR ~ 0-$1,500) among authors, who did not declare COI, but had one (Cohen’s d effect size estimate = 0.39, 95% CI: 0.77 − 0.02).

Means and medians of the length of time patients were followed after mesh implant surgery were reported in 48/56 studies. Median follow-up was 1.0 year, with a mean of 1.9 years. Follow-up duration was not associated with whether or not a study had at least one author with undeclared COI (*OR* = 0.82 95% CI:0.54 1.17). The small number of studies without COI (*n* = 3) precluded comparing follow-up duration between them and the 53 with COI.

The impact factors of the journals publishing studies were also examined to see if there was any relationship with number of undeclared COI. A journal’s impact factor did not predict whether or not a study had at least one author with undeclared COI (*OR* = 0.98 *95%CI*[0.75 1.3]).

There was a trend although no statistical association between being the lead or senior author and the presence of COI (*p* = 0.18). 65% of first authors had COI (declared or not), as did 56% of middle authors, and 69% of senior authors.

### Quality appraisal

We assessed the quality of each study using the 14 measures listed in Table 1. Only 26% (*n* = 14) of articles included a comparison group, partially reflecting the different study designs included in the review, and of those, 40% had comparable patients (e.g., age) in the intervention and control groups. The majority of studies (80%) did identify at least one patient characteristic such as age or obesity that could affect the success of mesh as a treatment. Only 28% (*n* = 13) of these studies, however, utilized these data in their analyses. The majority of publications explicitly stated that mesh was safe and beneficial (*n* = 32, 57%) although only 39% (*n* = 22) of all articles’ methods and outcomes supported these conclusions (Table 1). The small number of studies with no COI (3 of 56) precluded comparisons of quality between groups defined by the presence or absence of COI.

**Table 1:** Summary of measures of quality

Quality Dimension	Yes (%)^a^
a) Was there a comparison group?	14/53 (26)
b) Were the two groups similar and recruited from the same population?	6/14 (43)
c) Were the exposures measured similarly to assign people to both exposed and unexposed group?	7/11 (64)
d) Was at least one other patient factor identified? (e.g., age, obesity, co-morbidities)	45/56 (80)
e) Were strategies to deal with these factors stated?	13/46 (28)
f) Was there objective clinical evidence of POP or SUI (i.e., did the patents have symptoms of POP or SUI)?	35/52 (67)
g) Was there an a priori statement of benefit?	17/51 (33)
h) Did outcomes include objective and subjective measures using validated tools?	33/54 (61)
i) Was follow-up time reported and what was it?	46/54 (85)
j) Was follow-up complete, and if not, were the reasons to loss to follow-up described and explored?	23/48 (48)
k) Were appropriate statistical analyses used?	42/53 (79)
l) Did the authors make a statement that mesh was safe to use?	32/56 (57)
m) Were the inclusion/exclusion criteria fair? (i.e., authors did not appear to be including or excluding patients inappropriately)	27/55 (49)
n) Are the authors’ conclusions supported by the findings?	22/56(39)

^a^Denominators vary as not all quality dimensions could be assessed in all studies. For example, if there were no comparison group then questions b and c were not applicable

## Discussion

95% of the 56 articles reviewed had at least one author among those who could be assessed using Open Payments who received industry funding. The majority of this funding (47/53 of articles) was undeclared. COI among American MD authors studying pelvic mesh are substantial (60%), and most (81%) are undeclared. This level of unacknowledged industry support aligns with findings of a meta-analysis of studies of undisclosed industry support to physicians in general [7] and of clinical practice guideline authors’ COI [23]. It may also explain why, despite patient reports and legal findings of harm, the scholarly literature tends to endorse pelvic mesh as effective and safe.

In 2009, the International Committee of Medical Journal Editors (ICMJE) introduced requirements for detailed disclosure of all relevant COI by any author [24]. All articles in this review were published well after this. Observed non-compliance could arise from journal laxity, researchers’ sense of impunity, conviction that they are not swayed by industry largess, or convincing themselves that funding received was not related to the reported research. 36% of all authors received undeclared industry support of less than $1000. Some might consider that smaller levels of funding which may not have been offered explicitly for research are unlikely to sway physicians and should, therefore, be exempt from required reporting. In reality, even small gifts and gratuities have repeatedly been found to ‘win over’ physicians’ research and practice [7]. In our study, industry-funding had an equivocal impact on research quality and reported outcomes. The majority of publications explicitly stated that mesh was safe and beneficial (57%, *n* = 32) although only 10 of those 32 substantiated this with evidence. The median follow-up time of one-year post-op would have missed long-term complications. Such complications and failures of pelvic mesh are known to arise years after its insertion. For this reason, follow-up duration was chosen as a key indicator of study validity. As most studies were retrospective chart reviews longer follow-up duration could have been built into research designs. Indicators of poor research quality did not vary with authors’ declarations of industry support. The near ubiquitous presence of industry funding, however, precluded assessment of quality differences in articles with and without COI, and left us unable to really address aim 3 of this study.

### Limitations

The ability to track COI of all authors rather than only US physicians would help clarify the full extent and impact of industry funding on study design, findings, and interpretation of results. Open Payments data only include physicians licenced in the US. The database is verified and frequently updated but does not presume to include all payments made [25]. Accurate tracking of funding is further compromised because device manufacturers are known to violate reporting requirements [26]. Payments made to researchers’ family members, research or office staff, PhDs, institutions rather than individuals, etc., and any payments originating outside the US cannot, at present, be tracked. By extracting payment information for the year preceding, the year of and the year after publication we have attempted to identify all payments relevant to the articles studied, but may have missed some industry funding for included studies or captured funding for unrelated projects. It is also possible that funding received was not linked to the reviewed publication. Journal non-compliance with ICMJE requirements for declaring COI may have removed the reporting requirement for some authors and some funding. The overall impact of all these limitations may be an underestimation of the extent of undeclared industry funding to researchers.

Although we attempted to standardize our appraisal of articles, quality appraisal, as the name suggests, involves qualitative elements. The authors first rated each article separately then engaged in discussion to reach consensus, but acknowledge that the ‘objectivity’ of this process could be questioned.

## Conclusions

Industry funding for medical research is, at present, substantial and can be a source of innovation, but needs to also be ethical and transparent. During the timeframe studied the extent of industry involvement in research explicitly justifying the merit of pelvic mesh was high, while findings were at odds with concurrent FDA warnings of risk [14]. Equally important, self-reporting of financial COI by researchers appears to be unreliable and often contravenes requirements agreed upon by international medical journal editors. Industry funding both declared and, to a greater extent, undeclared, permeates almost all research on pelvic mesh and almost certainly shapes the quality of and conclusions drawn from those studies. This biased evidence in turn skews the risk benefit picture and potentially drives overuse of pelvic mesh in clinical practice.

## Data Availability

All data used and generated can be made available by the corresponding author upon reasonable request.

## Abbreviations

CMS Open Payments: United States Centers for Medicare & Medicaid Services Open Payments
COI: Conflicts of interest
FDA: US Food and Drug Administration
ICMJE: International Committee of Medical Journal Editors
IQR: Interquartile range
MD: Medical doctor
OR: Odds ratio
POP: Pelvic organ prolapse
SUI: Stress urinary incontinence
